# Li–fluorine codoped electrospun carbon nanofibers for enhanced hydrogen storage†

**DOI:** 10.1039/d0ra06500e

**Published:** 2021-01-20

**Authors:** Xiaohong Chen, Zhiyong Xue, Kai Niu, Xundao Liu, Wei lv, Bao Zhang, Zhongyu Li, Hong Zeng, Yu Ren, Ying Wu, Yongming Zhang

**Affiliations:** Institute of Advanced Materials, North China Electric Power University Beijing China yingwu2000@hotmail.com; School of Chemistry and Chemical Engineering, Shanghai Jiao Tong University No. 800 Dongchuan Rd., Minhang District Shanghai 200240 China ymzsjtu@gmail.com; School of Materials Science and Engineering, University of Jinan Jinan 250022 China

## Abstract

Carbon materials have attracted increasing attention for hydrogen storage due to their great specific surface areas, low weights, and excellent mechanical properties. However, the performance of carbon materials for hydrogen absorption is hindered by weak physisorption. To improve the hydrogen absorption performance of carbon materials, nanoporous structures, doped heteroatoms, and decorated metal nanoparticles, among other strategies, are adopted to increase the specific surface area, number of hydrogen storage sites, and metal catalytic activity. Herein, Li–fluorine codoped porous carbon nanofibers (Li–F–PCNFs) were synthesized to enhance hydrogen storage performance. Especially, perfluorinated sulfonic acid (PFSA) polymers not only served as a fluorine precursor, but also inhibited the agglomeration of lithium nanoparticles during the carbonization process. Li–F–PCNFs showed an excellent hydrogen storage capacity, up to 2.4 wt% at 0 °C and 10 MPa, which is almost 24 times higher than that of the pure porous carbon nanofibers. It is noted that the high electronegativity gap between fluorine and lithium facilitates the electrons of the hydrogen molecules being attracted to the PCNFs, which enhanced the hydrogen adsorption capacity. In addition, Li–F–PCNFs may have huge potential for application in fuel cells.

## Abstract 2

We developed a facile, yet general, approach for preparing Li–fluorine codoped porous carbon nanofiber (Li–F–PCNF) composites, which showed excellent hydrogen storage performance.

## Graphical abstract

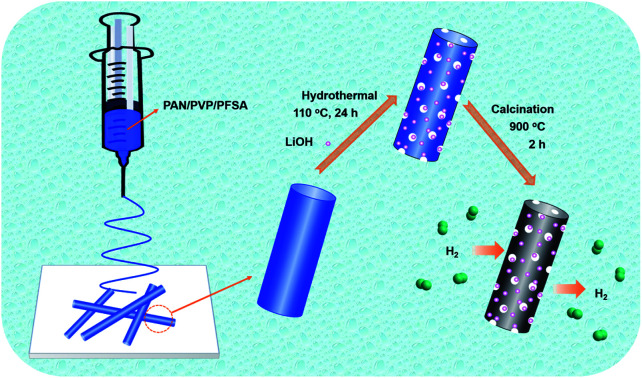

## Introduction

Hydrogen has attracted increasing attention as an environmentally safe energy source, and with great potential for application in powered vehicles.^1,2^ In recent decades, significant efforts to find new hydrogen storage materials have been made, such as metal alloys, complex hydrides, metal–organic frameworks, carbon materials, *etc.*^3–15^ Interestingly, carbon-based materials, with their low cost and weight, have long been considered as suitable adsorption substrates for the reversible storage of hydrogen. Yang reported that the hydrogen gas uptake of a multiwalled carbon nanotube is only 0.21 wt% at 77 K and 1 bar.^16^ Furthermore, Bai showed that the maximum hydrogen storage of carbon nanofibers is 0.65 and 0.6 wt% H_2_ respectively, at 330 K and 9 MPa.^17,18^ However, a series of carbon-based materials (such as carbon fibers, graphene and carbon nanotubes) show a moderate performance,^19–23^ which is attributed to the weak physisorption between molecular hydrogen and carbon-based materials.^24–26^

To increase the physisorption-based storage capability, large surface areas,^27,28^ heteroatom doping^29–34^ and alkali metal loading^35–38^ of carbon-based materials are highly desired. High specific surface areas and appropriate pore sizes significantly improve hydrogen molecule adsorption, which could be fulfilled by nanoporous carbon structures.^39^ Kou's group focused on issue-oriented schemes for activating the electrospun CNFs in terms of enhancing the conductivity, modulating the pore configuration, and doping with heteroatoms, among others, in close reference to some applications in supercapacitors and hydrogen storage.^40–46^ Moreover, Cheng reported fluorine doped graphite intercalation compounds enhanced hydrogen adsorption, because of the donation of electron density from the fluorine atom to the H_2_ antibonding σ* orbital.^47–50^ Meanwhile, Pinkerton showed a high hydrogen absorption capacity of 1.3 wt%, when alkali metals were introduced into carbon materials.^51^ Using porous carbon materials, heteroatoms or alkali metals alone, the hydrogen storage capacity has been limited. Therefore, heteroatoms and alkali metals were co-introduced to porous carbon-based hydrogen storage materials to improve the hydrogen adsorption capacity.

Herein, we explore an easy and facile strategy to obtain Li–fluorine codoped porous carbon nanofibers (Li–F–PCNFs) for a higher hydrogen adsorption, prepared through electrospun, hydrothermal and calcination methods. More interestingly, Li–F–PCNFs not only afford more defect sites, but support fluorine and uniformly dispersed alkali metal dopants. Thus, the excellent hydrogen storage capacity of Li–F–PCNFs is expected to be obtained by the synergistic effect among these three factors.

## Experimental

### Materials

Polyvinylpyrrolidone (PVP; *M*_w_ = 300 000) was bought from Aldrich Co., Ltd. Polyacrylonitrile (PAN; *M*_w_ = 1 500 000) was purchased from J&K scientific Chemical Co., Ltd. Lithium hydroxide (LiOH) was gotten from Alfa Aesar Co., Ltd. Perfluorinated sulfonic acid (PFSA) resin solution (IEC = 1.1) was obtained from Dongyue Co., Ltd. Ethanol was gotten from Shanghai Lingfeng Chemical Reagent Co. Ltd. *N*,*N*-Dimethylformamide (DMF) was provided by Sinopharm Chemical Reagent Co., Ltd. Those solvents were all of analytical grade and used as received.

### Apparatus

The morphology of the PCNFs, F–PCNFs, and Li–F–PCNFs composites was characterized by transmission electron microscopy (TEM) and scanning electron microscopy (SEM) using JEM-2010HT and JEOL2100F (both from Electron Optics Laboratory Co., Ltd., Japan), respectively. FT-IR was recorded on a Perkin Elmer spectrum 100 FTIR spectrometer. Powder X-ray diffraction (XRD) analyses were performed on a diffractometer (Bruker, German, APLX-DUO) with Cu Kα radiation. Raman spectra were recorded using a Thermo Fisher H31XYZE-US with an excitation wavelength of 532 nm. The specific surface area of the sample was calculated by the Brunauer–Emmett–Teller (BET) equation (Quantachrome Instruments version 11.02, America). Hydrogen storage properties were tested by a full-automatic PCI monitor apparatus (Suzuki, Japan, Shokan Co., Ltd.)

### Synthetic procedures

#### Preparation of porous carbon nanofibers (PCNFs) and F-doped porous carbon nanofibers (F–PCNFs)

In the DMF solvent, PAN, PVP and PFSA were added to furnish a content of 5%w/w, 5%w/w and 0.1%w/w, respectively. Then the mixture was electrospun in electric fields of the order of 1 kV cm^−1^. Afterwards, the PAN/PVP/PFSA nanofibers were transferred into a 100 mL Teflon stainless steel autoclave, then deionized water added. The hydrothermal treatment was conducted at 110 °C for 24 h to remove PVP. Porous PAN/PFSA nanofibers were obtained after drying at 80 °C in an oven. Furthermore, stabilization and carbonization of the above porous PAN/PFSA nanofibers, which were placed in the center of a quartz tube under argon flow, were completed in a high-temperature furnace.^52,53^ With argon flow for 25 min, the porous PAN/PFSA nanofibers were heated to 900 °C and annealed for 2 h, then cooled to room temperature to yield the final 0.1F–PCNFs. Meanwhile, 0.01F–PCNFs and 0.05F–PCNFs were obtained according to the content of PFSA with 0.01 wt% and 0.05 wt%, respectively.

For comparison, pristine PCNFs were also fabricated under the same conditions, without PFSA in the precursor solution. In addition, LiOH (2 wt%) was added to the hydrothermal treatment, and a series of Li–F–PCNFs composites (0.01Li–F–PCNFs, 0.05Li–F–PCNFs and 0.1Li–F–PCNFs) was finally obtained according to the above F–PCNFs procedure.

### Hydrogen storage capability measurements

The hydrogen adsorption properties of the samples, with a typical amount of around 150 mg, were conducted in a conventional Sieverts-type apparatus (Suzuki, PCT-1SPWIN, Japan). The hydrogen adsorption rate measurements were performed at 0 °C with a pressure of 10 MPa. Before the measurements, the samples were degassed under vacuum at 300 °C for at least 10 h.

## Results and discussion

**Fig. 1:**
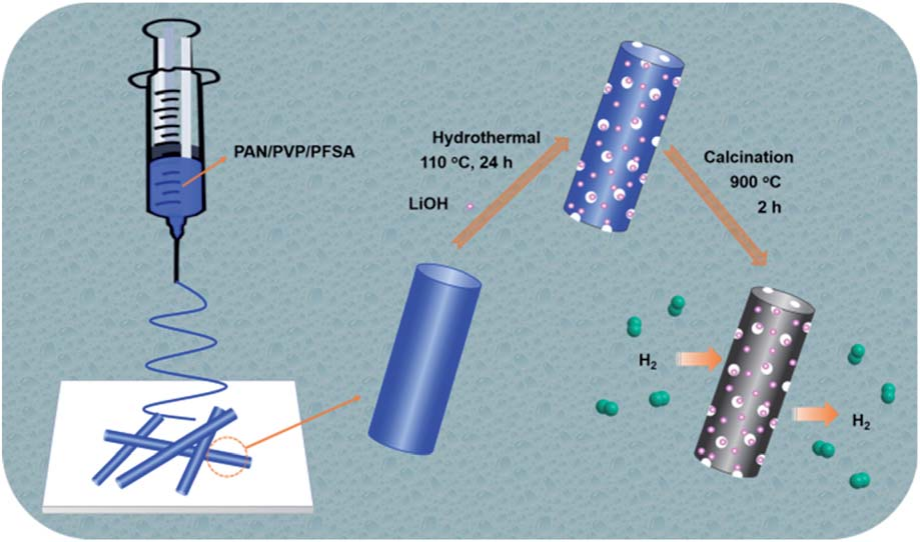
The fabrication procedure for the Li–F–PCNFs composites.

The overall synthetic procedure for the Li–F–PCNFs composites is illustrated in Fig. 1. PAN/PVP/PFSA nanofibers were first obtained *via* electrospinning of the mixture of PAN, PVP and PFSA in DMF solution. After removing PVP and exchanging the Li ion by a hydrothermal method and then drying, porous PAN/PFSA–Li nanofibers were preoxidized and carbonized at 220 and 900 °C under argon, respectively, yielding the resulting Li–F–PCNFs composites.

**Fig. 2:**
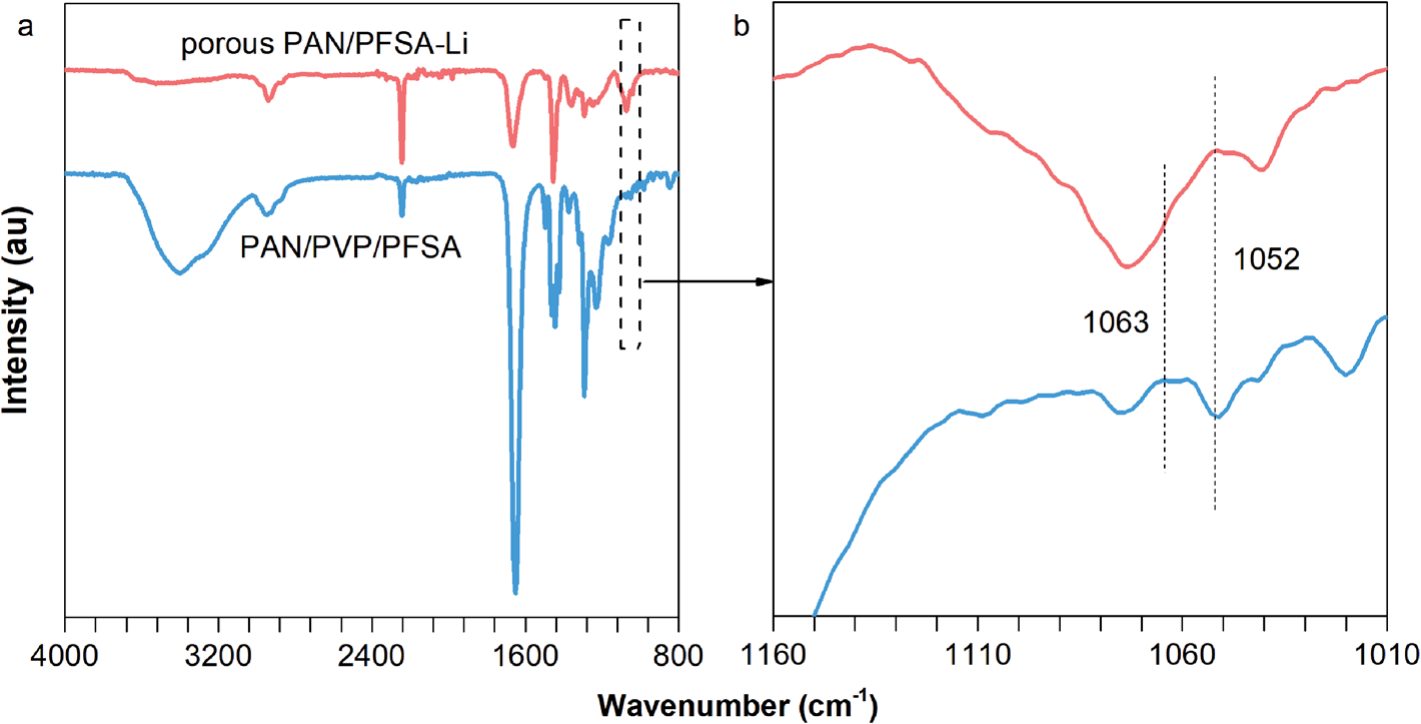
(a) FT-IR spectra and (b) a magnified view of the spectra of PAN/PVP/PFSA and porous PAN/PFSA–Li.

In order to validate that the –SO_3_H successfully converted to –SO_3_Li from PFSA, FT-IR spectra of the PAN/PVP/PFSA and porous PAN/PFSA–Li nanofibers were conducted and are shown in Fig. 2. The characteristic peak of sulfonate (–SO_3_H) groups at 1052 cm^−1^ of PAN/PVP/PFSA is absent after hydrothermal treatment. Meanwhile, the emerging peak at 1063 cm^−1^ associated with –SO_3_–Li is observed, indicating SO_3_–H successfully converted into the SO_3_–Li of PFSA.^54,55^

**Fig. 3:**
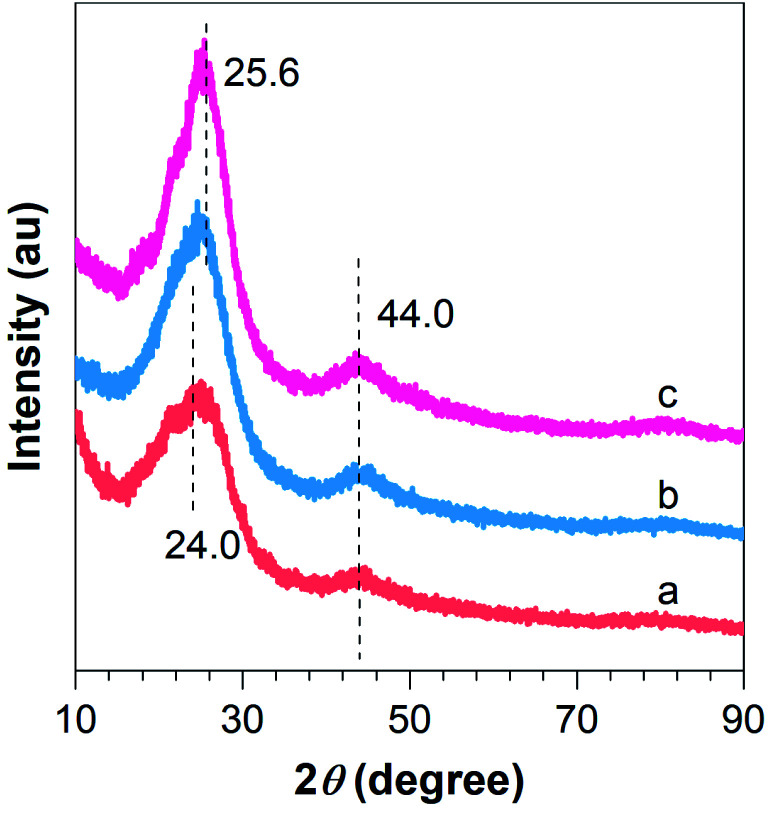
XRD patterns of (a) PCNFs, (b) 0.1F–PCNFs, and (c) 0.1Li–F–PCNFs composites.

To further demonstrate the successful preparation of the composites, XRD analysis was conducted. As can be seen from Fig. 3a, two broad peaks located at around 24.0 and 44.0° are evident for F–CNFs, which can be attributed to the graphite (002) and (004) crystalline plane with a hexagonal structure. Furthermore, with fluorine doping and lithium incorporation, the broad diffraction peak at 24.0° became redshifted to a sharp diffraction peak at 25.6°, indicating the graphitization degree enhancement of the 0.1Li–F–PCNFs composites, which is caused by the formation of Li nanoparticles (Li NPs) and fluorine doping on the surface of PCNFs (Fig. 3b and c). Meanwhile, the diffraction peaks from 0.01F–PCNFs, 0.05F–PCNFs, 0.01Li–F–PCNFs, and 0.05Li–F–PCNFs composites show a similar phenomenon, which further verifies the cooperation among fluorine and lithium factors (Fig. S1†). This is evident from the diffraction pattern of the resulting Li–F–PCNFs composites. However, the weak signals of Li NPs are most likely because of its low crystallinity.

**Fig. 4:**
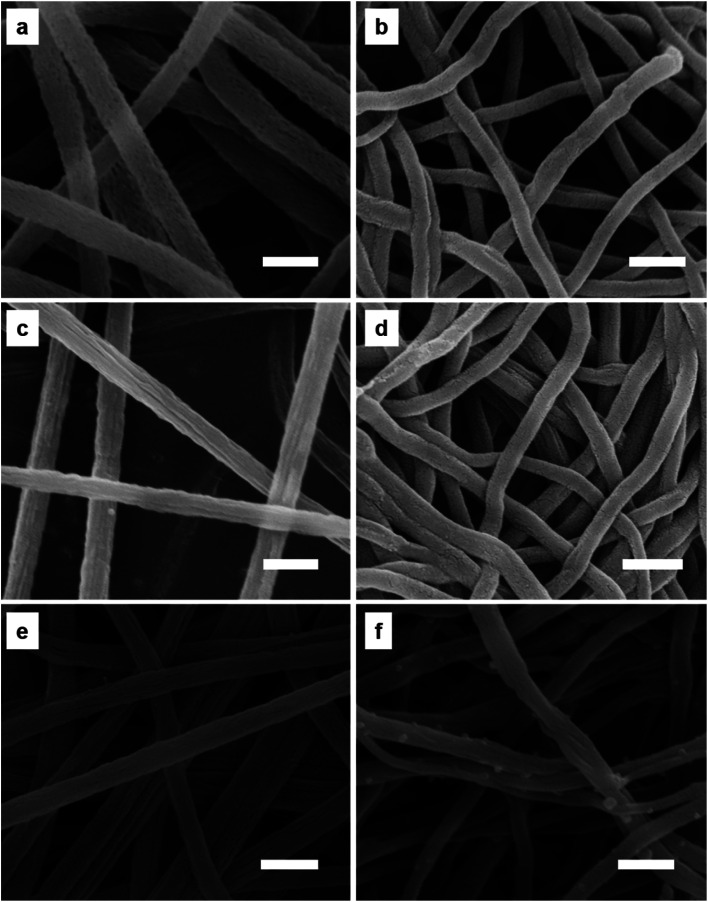
SEM images of (a) porous PAN, (b) PCNFs, (c) porous PAN/PFSA, (d) 0.1F–PCNFs, (e) 0.1Li–porous PAN/PFSA, and (f) 0.1Li–F PCNFs composites. The scale bar is 500 nm.

Morphologies of PCNFs and their corresponding composites were further revealed by SEM observation. PAN/PVP and PAN/PVP/PFSA nanofibers exhibit smooth surfaces (Fig. S2, S3 and S6†), after removing PVP through a hydrothermal method. Porous PAN and PAN/PFSA nanofibers show many nanopores and rough surfaces (Fig. 4a and c, S4 and S7†). Meanwhile, some LiOH nanoparticles are also evenly dispersed on the porous PAN/PFSA–Li surface, after the ion exchange between Li^+^ and H^+^ ions of PFSA (Fig. 4e, S9 and S11†). After being carbonized, PCNFs and F–PCNFs exhibit rough surfaces with diameters ranging from 150 to 250 nm (Fig. 4b and d, S5 and S8†), such nanopores can provide more active sites for further alkali metal nanoparticles deposition and high hydrogen storage.

When deposited on the F–PCNFs, Li NPs are relatively homogeneously dispersed (Fig. 4f, S10 and S12†) on the surface of PCNFs with the diameter ranging from 160 to 270 nm (Fig. 4e). Such a greatly improved homogeneous distribution of Li NPs might result from the rough surface of F–PCNFs and ion bonding between sulfonate and alkali metal ions, which can provide more nucleation sites for the formation and also prevent the agglomeration of Li NPs, and result in an improvement in the hydrogen storage performance. Furthermore, HRTEM images (Fig. S13a and b†) show that nanopores are uniformly dispersed on the surface of PCNFs and also display that the Li–F–PCNFs composites have a diameter of about 200 nm, and energy dispersive spectroscopy (EDS) spectrum (Fig. S13c†) also indicates the presence of C, O, N, F and S elements, which is consistent with the above SEM results. Generally, the EDS of HRTEM cannot be useful for lithium. There is no lithium element in the EDS of 0.1Li–F–PCNFs.

The nitrogen adsorption–desorption isotherm and the corresponding Barrett–Joyner–Halenda (BJH) pore size distribution curve of the obtained 0.1Li–F–PCNFs composites were measured. The specific surface area of 0.1Li–F–PCNFs composites is 34.3 m^2^ g^−1^. Obvious adsorption steps from 0.1Li–F–PCNFs composites can be found in the relative pressure range of 0.4–1.0 MPa. Moreover, the pore sizes analyzed by the BJH method show that the primary mesopore size is 2.65 nm for the 0.1Li–F–PCNFs composites (Fig. S14†). These can be ascribed to the capillary condensation of nitrogen molecules in mesopores with relatively uniform dimensions,^56^ indicating the end of the capillary condensation and narrow pore size distribution, which has a beneficial effect on increasing hydrogen absorption.

**Fig. 5:**
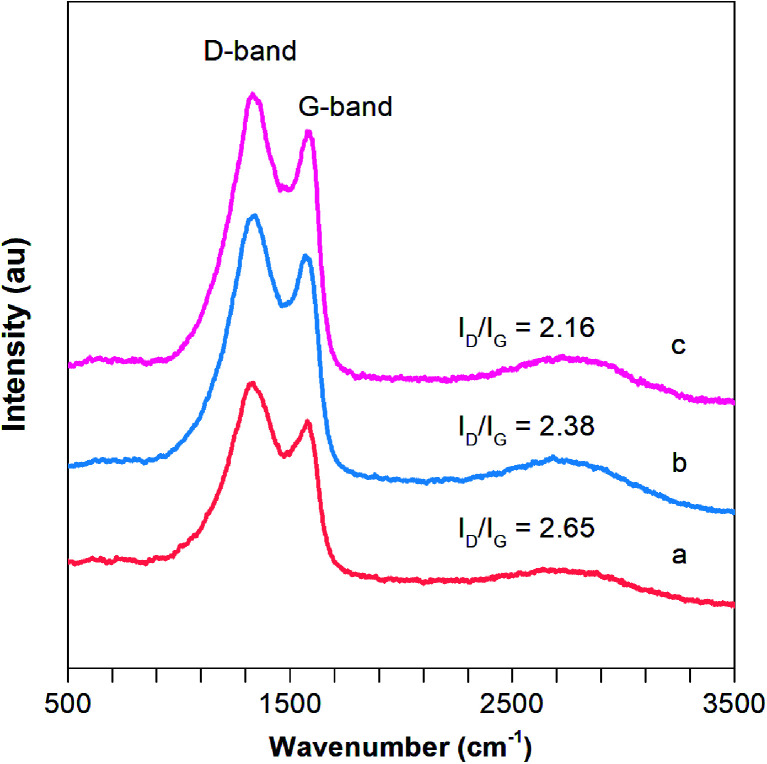
Raman spectra of (a) 0.01Li–F–PCNFs, (b) 0.05Li–F–PCNFs, and (c) 0.1Li–F–PCNFs composites.

Raman spectroscopy is a useful tool for the characterization of the carbon structure of porous CNFs based materials. As depicted in Fig. 5, two strong bands at around 1347 and 1586 cm^−1^ are observed in 0.01F–PCNFs, 0.05F–PCNFs, 0.1F–PCNFs, 0.01Li–F–PCNFs, 0.05Li–F–PCNFs and 0.1Li–F–PCNFs composites, which correspond to the disordered (D band, defects) and graphitic carbons (G band for sp^2^ domains), respectively. It is noted that the area ratio of the D band to the G band (*I*_D_/*I*_G_) for 0.1Li–F–PCNFs is 2.16, which is much lower than those of 0.01F–PCNFs (3.04), 0.05F–PCNFs (2.56), 0.1F–PCNFs (2.26), 0.01Li–F–PCNFs (2.65) and 0.05Li–F–PCNFs (2.38) (Fig. 5b and c and S15†). These results indicate the greatly enhanced graphitic structure of the former, which might be mainly attributed to the graphitization effect of fluorine and lithium dopants. Such an improved graphitic structure can offer an enhanced carbon framework sp^2^ conjugation of PCNFs surfaces, which is favorable for further hydrogen absorption processes.

**Fig. 6:**
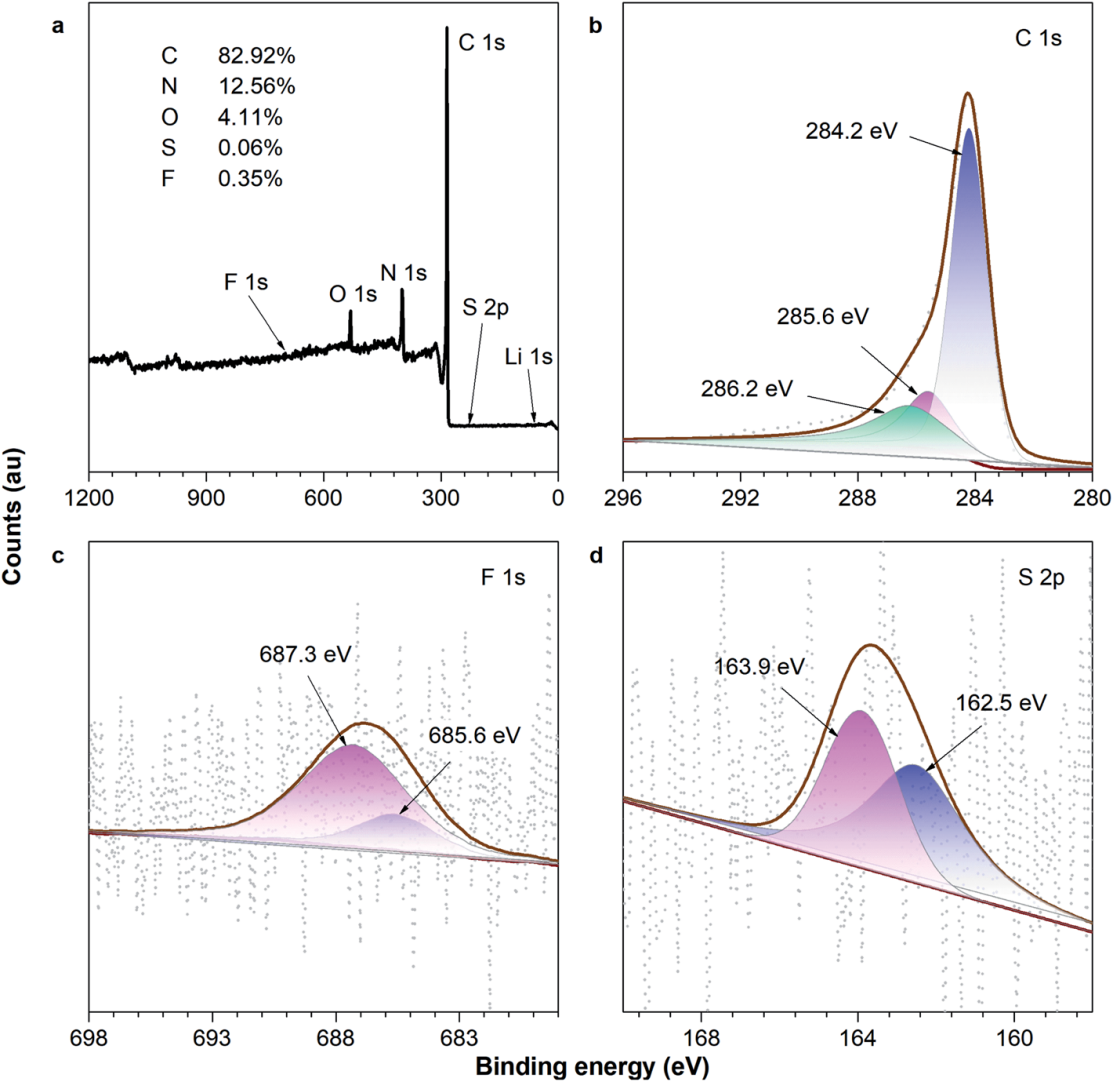
XPS spectra: (a) survey scan; and the (b) C 1s, (c) F 1s and (d) S 2p regions of the 0.1Li–F–PCNFs composites.

In order to obtain more information on the 0.1Li–F–PCNF after the codoping processes, the composite was analyzed by XPS. Fig. 6 shows the XPS survey together with the high resolution XPS spectra of F 1s and Li 1s. Fig. 6a clearly displays intense F 1s and Li 1s peaks, thus verifying the successful codoping of PCNFs with surface components of 82.92% C, 12.56% N, 4.11% O, 0.06% S, and 0.35% F. There is a peak at 284.2 eV of C 1s, indicating pure carbon, and 286.2 eV, related to C–F bond. Meanwhile, the peak at 285.6 eV of C 1s is related to C–N, C–O and C–S bonds, which may be introduced from PVP and lithium hydroxide residuals. Furthermore, it is evident from the F 1s deconvoluted peaks (Fig. 6c) that fluorine is incorporated into the CNFs in two configurations: C–F (685.6 eV) and C–F (687.3 eV) fluorine species,^57–59^ which indicate that the fluorine element is successfully doped into the PCNFs. Meanwhile, it is evident from the S 2p deconvoluted peaks (Fig. 6d) that sulfur is incorporated into the PCNFs in two configurations: the thiophene (S–C–S) (162.5 eV), and oxidized (SO_*x*_–C) (163.9 eV) sulfur species. This is attributed to a higher positive charge and spin density of carbon atoms, which can provide the more active sites and offer a better hydrogen adsorption density. Notably, the theoretical atom weight content of lithium is calculated as 0.013 wt% (the molar ratio of S : Li = 1), which is too low to be accurately determined.

**Fig. 7:**
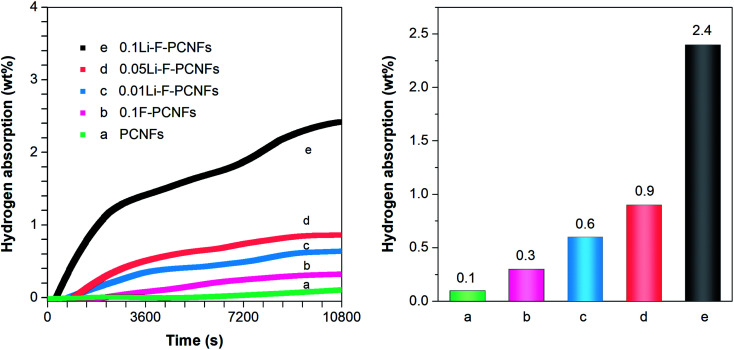
Hydrogen adsorption spectra (left) and capacities (right) of (a) PCNFs, (b) 0.1 F–PCNFs, (c) Li–0.01F–PCNFs, (d) Li–0.05F–PCNFs, and (e) Li–0.1F–PCNFs composites at 0 °C under a hydrogen pressure of 10 MPa.

The hydrogen adsorption experiments were carried out under 10 MPa at 0 °C. Typical high-pressure hydrogen adsorption isotherms are shown in Fig. 7. This shows that the hydrogen concentration gradually increases as the adsorption time increases. The adsorption performance of PCNFs and 0.1F–PCNFs composites is almost complete within 10 800 s, with storage capacities of 0.1 and 0.3 wt%, respectively. This illustrates that F–PCNFs can enhance the hydrogen adsorption, compared to PCNFs composites. In order to further increase the hydrogen storage capacity, alkali metal lithium was introduced into the F–PCNFs. Moreover, those sulfonate ions from PFSA can be exchanged with lithium hydroxide to anchor the lithium ions, so that they are fixed during the carbonization process of porous PAN/PFSA–Li, thereby obtaining lithium particles dispersed uniformly on the surface of F–PCNFs. As shown in Fig. 7c–e, the hydrogen storage capacities of 0.01Li–F–PCNFs, 0.05Li–F–PCNFs and 0.1Li–F–PCNFs are about 0.6, 0.9 and 2.4 wt%, respectively, under the above measurement conditions, which are enhanced with the increasing contents of fluorine and lithium dopants. This is attributed to the high electron affinity of the sp^2^ carbon framework resulting from fluorine and lithium dopants, providing strong stabilization among Li–F–PCNFs and the molecular H_2_. More importantly, lithium dopants act as positive (acidic) cores that attract hydrogen molecules. The above-mentioned results suggest that fluorine and lithium dopants in PCNFs endow these composites with an excellent hydrogen storage performance, which could reach the DOE goals in the future.

**Fig. 8:**
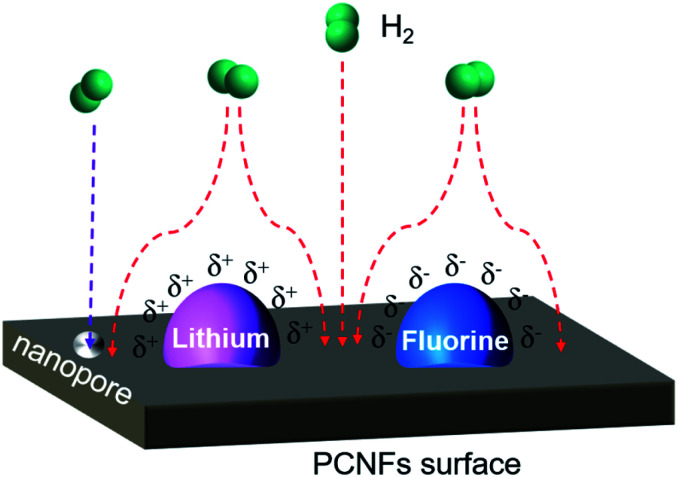
The mechanism of hydrogen adsorption by the Li–F–PCNFs composites.

The mechanism of hydrogen storage from Li–F–PCNFs is shown in Fig. 8. When the hydrogen molecule moves from outside the surface of PCNFs, the hydrogen molecule is affected by the electronegativity gap between the lithium and fluorine. Fluorine has the highest electronegativity (4.0) among the elements and lithium has the lowest electronegativity (0.98). The electronegativity of carbon lies between that of fluorine and lithium at 2.55. Therefore, fluorine exhibits electrically negative-charged characteristics (*δ*^−^) on the surface, and the lithium exhibits relatively positively charged characteristics (*δ*^+^). Consequently, this electronegativity difference will electrically attract the hydrogen molecule into PCNFs.^60^ The electrons from the hydrogen molecule are attracted towards the surface by electric force and can be adsorbed on the surface of the PCNFs more efficiently. Furthermore, the nanopore size of the PCNFs is 2.65 nm for the Li–F–PCNFs composites, which can be ascribed to the capillary condensation according to the equilibrium temperature of adsorption and the geometrical characteristics of the adsorbent. Therefore, these effects are beneficial to an improvement of hydrogen storage.

## Conclusions

In summary, we report a facile synthesis procedure for the preparation of Li–F–PCNFs composites with noticeable performance as hydrogen storage materials. The Li–F–PCNFs were synthesized through a simple electrospinning, hydrothermal treatment, and carbonization process. Especially, PFSA was not only a fluorine source to enhance the hydrogen adsorption density, but it also acted to prevent the agglomeration of lithium nanoparticles through an ion-exchange method. The porous carbon framework endows the Li NPs with a uniform distribution, effective space confinement, and a much higher specific surface area. The unique architecture of the Li–F–PCNFs composites allows 2.4 wt% absorption under 10 MPa at 0 °C. Therefore, this strategy represents a highly novel approach for synthesizing Li–F–PCNF scomposites to act as hydrogen storage materials that exhibit superior storage properties. It is also expected that these materials can be significant potential materials in the electrochemical and fuel-cell fields.

## Author contributions

This work was finished through contributions from all authors. The design and synthesis of Li–F–PCNFs used in this work, and most of tests relating to the structure and morphology, were contributed to by Xiaohong Chen, Zhiyong Xue, Kai Niu, and Xundao Liu. With the help of Wei Lv, Bao Zhang, Zhongyu Li, Hong Zeng, and Yu Ren, the hydrogen storage experiments were successfully completed. Prof. Ying Wu and Prof. Yongming Zhang directed the whole process relating to this carbon-based storage system and revised the manuscript. All authors have given approval to the final version of this manuscript.

## Conflicts of interest

All authors declare that there are no conflicts of interest.

## Supplementary Material

RA-011-D0RA06500E-s001

## References

[cit1] Firlej L., Pfeifer P., Kuchta B. (2013). Understanding universal adsorption limits for hydrogen storage in nano porous systems. Adv. Mater..

[cit2] Nishihara H., Kyotani T. (2012). Templated nanocarbons for energy storage. Adv. Mater..

[cit3] Mortazavi S. Z., Parvin P., Reyhani A., Malekfarc R., Mirershadid S., Mohan M., Sharma V. K., Kumar E. A., Gayathri V. (2013). Hydrogen storage property of laser induced Pd-nanoparticle decorated multi-walled carbon nanotubes. RSC Adv..

[cit4] Xia G., Li D., Chen X., Tan Y., Tang Z., Guo Z., Liu H., Liu Z., Yu X. (2013). Carbon-coated Li_3_N nanofibers for advanced hydrogen storage. Adv. Mater..

[cit5] Schneemann A., White J. L., Kang S., Jeong S., Wan L. F., Cho E. S., Heo T. W., Prendergast D., Urban J. J., Wood B. C., Allendorf M. D., Stavila V. (2018). Nanostructured metal hydrides for hydrogen storage. Chem. Rev..

[cit6] Xue J., Wu T., Dai Y., Xia Y. (2019). Electrospinning and electrospun nanofibers: methods, materials, and applications. Chem. Rev..

[cit7] Wagemans R. W. P., van Lenthe J. H., de Jongh P. E., Dillen A. J., de Jong K. P. (2005). Hydrogen storage in magnesium clusters: quantum chemical study. J. Am. Chem. Soc..

[cit8] Zhang Y., Zhang H., Ding X., Liu D., Zhang Q., Si T. (2018). Microstructure characterization and hydrogen storage properties study of Mg_2_Ni_0.92_M_0.08_ (M = Ti, V, Fe or Si) alloys. Prog. Nat. Sci.: Mater. Int..

[cit9] Kang S. Y., Heo T. W., Allendorf M. D., Wood B. C. (2019). Morphology–dependent stability of complex metal hydrides and their intermediates using first-principles calculations. ChemPhysChem.

[cit10] Yuan W., Ge M., Wang K., Hou X., Liu N., Deng Z., Guo R., Liu S., Zhao Y., He J., Xi W., Luo J., Ding Y. (2019). Atomic-scale selectivity of hydrogen for storage sites in Pd nanoparticles at atmospheric pressure. Nanoscale.

[cit11] Chen J., Li S.-L., Tao Z.-L., Shen Y.-T., Cui C.-X. (2003). Titanium disulfide nanotubes as hydrogen-storage materials. J. Am. Chem. Soc..

[cit12] Ma Q., Yu Y., Sindoro M., Fane A. G., Wang R., Zhang H. (2017). Carbon-based functional materials derived from waste for water remediation and energy storage. Adv. Mater..

[cit13] Carr C. L., Jayawardana W., Zou H., White J. L., Gabaly F. E., Conradi M. S., Stavila V., Allendorf M. D., Majzoub E. H. (2018). Anomalous H_2_ desorption rate of NaAlH_4_ confined in nitrogen-doped nanoporous carbon frameworks. Chem. Mater..

[cit14] Zhang B., Yuan J., Wu Y. (2019). Catalytic effects of Mg(BH_4_)_2_ on the desorption properties of 2LiNH_2_–MgH_2_ mixture. Int. J. Hydrogen Energy.

[cit15] Zhang B., Wu Y. (2017). Recent advances in improving performances of the lightweight complex hydrides Li–Mg–NH system. Prog. Nat. Sci.: Mater. Int..

[cit16] Yang S. J., Cho J. H., Nahm K. S., Park C. R. (2010). Enhanced hydrogen storage capacity of Pt-loaded CNT@MOF-5 hybrid composites. Int. J. Hydrogen Energy.

[cit17] Hussain T., Mortazavi B., Bae H., Rabczukb T., Lee H., Karton A. (2019). Enhancement in hydrogen storage capacities of light metal functionalized borone graphdiyne nanosheets. Carbon.

[cit18] C Bai B., Kim G. J., Naik M., Lee Y.-S. (2011). The hydrogen storage capacity of metal-containing polyacrylonitrile-based electrospun carbon nanofibers. Carbon Lett..

[cit19] Cheng H., Pez G. P., Cooper A. C. (2001). Mechanism of hydrogen sorption in single-walled carbon nanotubes. J. Am. Chem. Soc..

[cit20] Zuttel A., Sudan P., Mauron P., Kiyobayashi T., Emmenegger C., Schlapbach L. (2002). Hydrogen storage in carbon nanostructures. Int. J. Hydrogen Energy.

[cit21] Chen X., Chi M., Xing L., Xie X., Liu S., Liang Y., Zheng M., Hu H., Dong H., Liu Y., Jiang S. P., Xiao Y. (2019). Natural plant template-derived cellular framework porous carbon as a high-rate and long-life electrode material for energy storage. ACS Sustainable Chem. Eng..

[cit22] Parambhath V. B., Nagar R., Ramaprabhu S. (2012). Effect of nitrogen doping on hydrogen storage capacity of palladium decorated graphene. Langmuir.

[cit23] Zhou H., Liu X., Zhang J., Yan X., Liu Y., Yuan A. (2014). Enhanced room-temperature hydrogen storage capacity in Pt-loaded graphene oxide/HKUST-1 composites. Int. J. Hydrogen Energy.

[cit24] Chen P., Wu X., Lin J., Tan K. L. (1999). High H_2_ uptake by alkali-doped carbon nanotubes under ambient pressure and moderate temperatures. Science.

[cit25] Fan Y. Y., Liao B., Liu M., Wei Y. L., Lu M. Q., Cheng H. M. (1999). Tailoring the diameters of vapor-grown carbon nanofibers. Carbon.

[cit26] Skowroński J. M., Scharff P., Pfänder N., Cui S. (2003). Room temperature electrochemical opening of carbon nanotubes followed by hydrogen storage. Adv. Mater..

[cit27] Woo Y., Kim B.-S., Lee J.-W., Park J., Cha M., Takeya S., Im J., Lee Y., Jeon T.-I., Bae H., Lee H., Han S. S., Yeo B. C., Kim D., Yoon J. (2018). Enhanced hydrogen-storage capacity and structural stability of an organic clathrate structure with fullerene (C_60_) guests and lithium doping. Chem. Mater..

[cit28] Carr C. L., Jayawardana W., Zou H., White J. L., Gabaly F. E., Conradi M. S., Stavila V., Allendorf M. D., Majzoub E. H. (2018). Anomalous H_2_ desorption rate of NaAlH_4_ confined in nitrogen-doped nanoporous carbon frameworks. Chem. Mater..

[cit29] Wang L., Yang R. T. (2009). Hydrogen storage properties of N-doped microporous carbon. J. Phys. Chem. C.

[cit30] Lan K., Gong L., Yang M., Huang X., Jiang P., Wang K., Ma L., Li R. (2019). Nitrogen and phosphorus dual-doping carbon shells encapsulating ultrafine Mo_2_C particles as electrocatalyst for hydrogen evolution. J. Colloid Interface Sci..

[cit31] Li B., Li J., Zhao H., Yu X., Shao H. (2019). Mg-based metastable nano alloys for hydrogen storage. Int. J. Hydrogen Energy.

[cit32] Liu B., Zhang B., Wu Y., Lv W., Zhou S. (2019). Theoretical prediction and experimental study on catalytic mechanism of incorporated Ni for hydrogen absorption of Mg. Int. J. Hydrogen Energy.

[cit33] Chong L., Zeng X., Ding W., Liu D.-J., Zou J. (2015). NaBH_4_ in “graphene wrapper:” significantly enhanced hydrogen storage capacity and regenerability through nanoencapsulation. Adv. Mater..

[cit34] Balahmar N., Mokaya R. (2019). Pre-mixed precursors for modulating the porosity of carbons for enhanced hydrogen storage: towards predicting the activation behaviour of carbonaceous matter. J. Mater. Chem. A.

[cit35] Bald C. P., Hereijgers B. P. C., Bitter J. H., de Jong K. P. (2006). Facilitated hydrogen storage in NaAlH_4_ supported on carbon nanofibers. Angew. Chem., Int. Ed..

[cit36] Tylianakis E., Psofogiannakis G. M., Froudakis G. E. (2010). Li-doped pillared graphene oxide: a graphene-based nanostructured material for hydrogen storage. J. Phys. Chem. Lett..

[cit37] Lee H., Ihm J., Cohen M. L., Louie S. G. (2010). Calcium-decorated graphene-based nanostructures for hydrogen storage. Nano Lett..

[cit38] Yang R. T. (2000). Hydrogen storage by alkali-doped carbon nanotubes – revisited. Carbon.

[cit39] Li Y., Xiao Y., Dong H., Zheng M., Liu Y. (2019). Polyacrylonitrile-based highly porous carbon materials for exceptional hydrogen storage. Int. J. Hydrogen Energy.

[cit40] Kou Z., Zang W., Pei W., Zheng L., Zhou S., Zhang S., Zhang L., Wang J. (2020). Sacrificial Zn strategy enables anchoring of metal single atoms on the exposed surface of holey 2D molybdenum carbide nanosheets for efficient electrocatalysis. J. Mater. Chem. A.

[cit41] Ma Y., Yang T., Zou H., Zang We., Kou Z., Mao L., Feng Y., Shen L., Pennycook S. J., Duan L., Li X., Wang J. (2020). Synergizing Mo single atoms and Mo_2_C nanoparticles on CNTs synchronizes selectivity and activity of electrocatalytic N_2_ reduction to ammonia. Adv. Mater..

[cit42] Kou Z., Zang W., Ma Y., Pan Z., Mu S., Gao X., Tang B., Xiong M., Zhao X., Cheetham A. K., Zheng L., Wang J. (2020). Cage-confinement pyrolysis route to size-controlled molybdenum-based oxygen electrode catalysts: from isolated atoms to clusters and nanoparticles. Nano Energy.

[cit43] Nie G., Zhao X., Luan Y., Jiang J., Kou Z., Wang J. (2020). Key issues facing electrospun carbon nanofibers in energy applications: ongoing approaches and challenges. Nanoscale.

[cit44] Dzenis Y. (2004). Spinning continuous fibers for nanotechnology. Science.

[cit45] Li J.-M. (2017). Realizing single-crystalline vertically-oriented and high-density electrospun nanofibril bundles by controlled postcalcination. CrystEngComm.

[cit46] Li J.-M., Wei D.-P., Hu Y.-B., Fang J., Xu Z.-A. (2014). Synthesis of ultrafine green-emitting BaCO_3_ nanowires with 18.5 nm-diameter by CO_2_ vapor-assisted electrospinning. CrystEngComm.

[cit47] Cheng H., Sha X., Chen L., Cooper A. C., Foo M.-L., Lau G. C., Bailey III W. H., Pez G. P. (2009). An enhanced hydrogen adsorption enthalpy for fluoride intercalated graphite compounds. J. Am. Chem. Soc..

[cit48] Nyulasi B., Kovacs A. (2006). Theoretical study of F–(H_2_)_*n*_ and Cl–(H_2_)_*n*_ (*n* = 1–8) anion complexes. Chem. Phys. Lett..

[cit49] Trewin A., Darling G. R., Cooper A. I. (2008). “Naked” fluoride binding sites for physisorptive hydrogen storage. New J. Chem..

[cit50] Liu X., Wu D., Liu X., Luo X., Liu Y., Zhao Q., Li J., Dong D. (2020). Perfluorinated comb-shaped cationic polymer containing long-range ordered main chain for anion exchange membrane. Electrochim. Acta.

[cit51] Pinkerton F. E., Wicke B. G., Olk C. H., Tibbetts G. G., Meisner G. P., Meyer M. S., Herbst J. F. (2000). Thermogravimetric measurement of hydrogen absorption in alkali-modified carbon materials. J. Phys. Chem. B.

[cit52] Pei S., Zhou Z., Chen X., Huang X., Liu T., Cao B., Wang F. (2016). Co/CoO nanoparticles/Ag nanowires/nitrogen codoped electrospun carbon nanofibers as efficient electrocatalysts for oxygen reduction. Int. J. Electrochem. Sci..

[cit53] Liu X., Luo X., Chen X., Zou S., Liu X., Li J., Li H., Dong D. (2020). Perfluorinated membrane electrode assembly containing metal-free-catalyst cathode for anion exchange membrane fuel cells. J. Electroanal. Chem..

[cit54] Falk M. (1980). An infrared study of water in perfluorosulfonate (Nafion) membranes. Can. J. Chem..

[cit55] Cai Z., Liu Y., Liu S., Li L., Zhang Y. (2012). High performance of lithium-ion polymer battery based on nonaqueous lithiated perfluorinated sulfonic ion-exchange membranes. Energy Environ. Sci..

[cit56] Xia K., Gao Q., Wu C., Song S., Ruan M. (2007). Activation, characterization and hydrogen storage properties of the mesoporous carbon CMK-3. Carbon.

[cit57] Normand F. L., Hommer J., Szörényi T., Fuchs C., Fogarassy E. (2001). XPS study of pulsed laser deposited CN_*x*_ films. Phys. Rev. B: Condens. Matter Mater. Phys..

[cit58] Tressaud A., Moguet F., Flandrois S., Chambon M., Guimon C., Nanse G., Papirer E., Gupta V., Bahl O. P. (1996). On the nature of C–F bonds in various fluorinated carbon materials: XPS and TEM investigations. J. Phys. Chem. Solids.

[cit59] Palchan I., Crespin M., Estarde-szwarckope H., Rousseau B. (1989). Graphite fluorides: an XPS study of a new type of C–F bonding. Chem. Phys. Lett..

[cit60] Im J. S., Park S.-J., Lee Y.-S. (2009). The metal–carbon–fluorine system for improving hydrogen storage by using metal and fluorine with different levels of electronegativity. Int. J. Hydrogen Energy.

