# Supplementary material for: Li–fluorine codoped electrospun carbon nanofibers for enhanced hydrogen storage
Source: RSC Adv. 2021 Jan 20;11(7):4053–61. doi: 10.1039/d0ra06500e (PMC8694184; doi:10.1039/d0ra06500e)
Supplement: RA-011-D0RA06500E-s001 [file RA-011-D0RA06500E-s001.pdf]

Electronic Supplementary Material (ESI) for RSC Advances.
This journal is © The Royal Society of Chemistry 2020

Supporting Information

Li-fluorine-codoped electrospun carbon nanofibers for enhanced hydrogen storage

Xiaohong Chen,[a] Zhiyong Xue,[a] Kai Niu,[b] Xundao Liu,[c] Wei Lv,[a] Bao Zhang,[a] Zhongyu Li,[a] Hong Zeng,[a] Yu Ren,[a] Ying Wu,*[a] and Yongming Zhang*[b]

[a] Institute of Advanced Materials, North China Electric Power University, Beijing

[b] School of Chemistry and Chemical Engineering, Shanghai Jiao Tong University, No. 800 Dongchuan Rd., Minhang District, Shanghai 200240, China. E-mail: ymzsjtu@gmail.com,

[c] School of Materials Science and Engineering, University of Jinan, Jinan, 250022 China.

E-mail: yingwu2000@hotmail.com; ymzsjtu@gmail.com

Contents

Fig. S1. XRD patterns of (a) 0.01F-PCNFs, (b) 0.05F-PCNFs, (c) 0.01Li-F-PCNFs and (d) 0.05Li-F-PCNFs composites

Fig. S2. SEM images of (a) PAN/PVP and (b) PAN/PVP/0.1PFSA.

Fig. S3. SEM images of PAN/PVP/0.01PFSA.

Fig. S4. SEM images of porous PAN/0.01PFSA.

Fig. S5. SEM images of 0.01F-PCNFs.

Fig. S6. SEM images of PAN/PVP/0.05PFSA.

Fig. S7. SEM images of porous PAN/0.05PFSA.

Fig. S8. SEM images of 0.05F-PCNFs.

Fig. S9. SEM images of porous PAN/0.01PFSA-Li.

Fig. S10. SEM images of 0.01Li-F-PCNFs composites.

Fig. S11. SEM images of porous PAN/0.05PFSA-Li.

Fig. S12. SEM images of 0.05Li-F-PCNFs composites.

Fig. S13. (a, b) HRTEM images and (c) EDS of 0.1Li-F-PCNFs composites.

Fig. S14. Nitrogen adsorption–desorption isotherms of 0.1Li-F-PCNFs composites obtained at 77 K.

Fig. S15. Raman spectra of (a) 0.01F-PCNFs composites, (b) 0.05F-PCNFs composites, and (c) 0.1F-PCNFs composites.

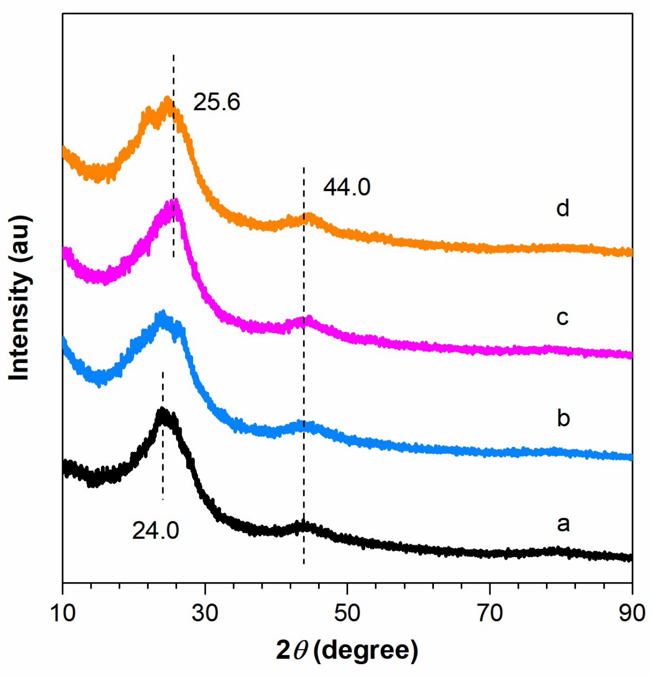

Fig. S1. XRD patterns of (a) 0.01F-PCNFs, (b) 0.05F-PCNFs, (c) 0.01Li-F-PCNFs and (d) 0.05Li-F-PCNFs composites

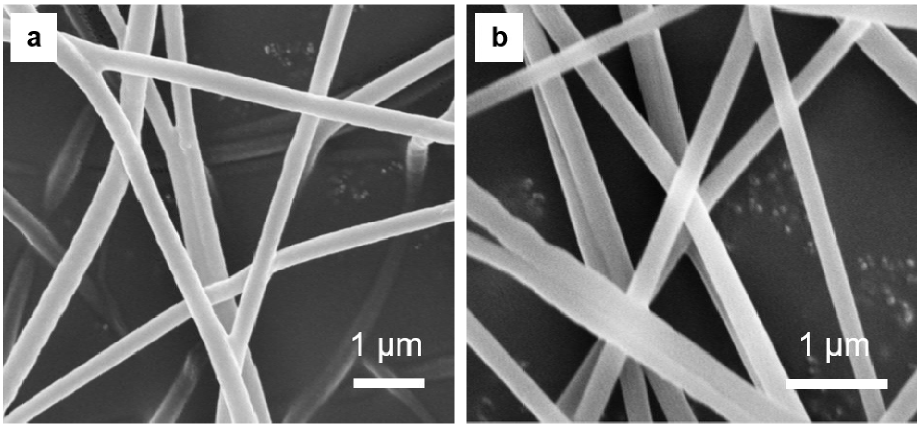

Fig. S2. SEM images of (a) PAN/PVP and (b) PAN/PVP/0.1PFSA.

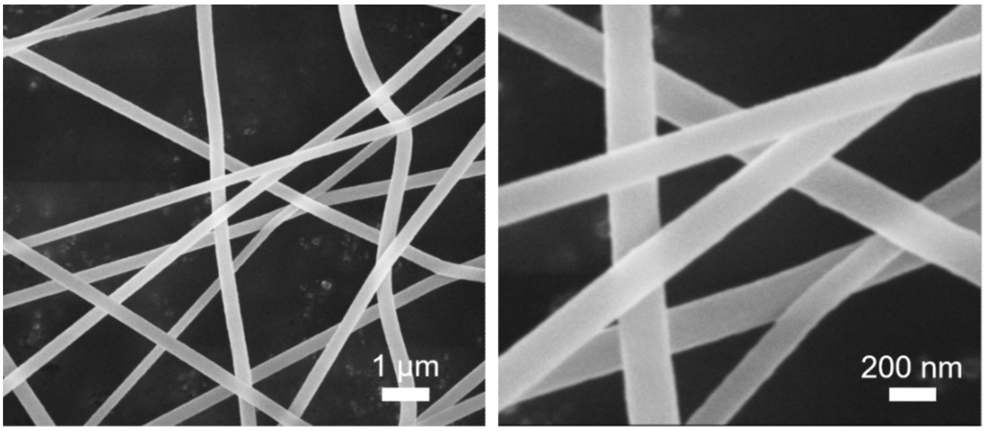

Fig. S3. SEM images of PAN/PVP/0.01PFSA.

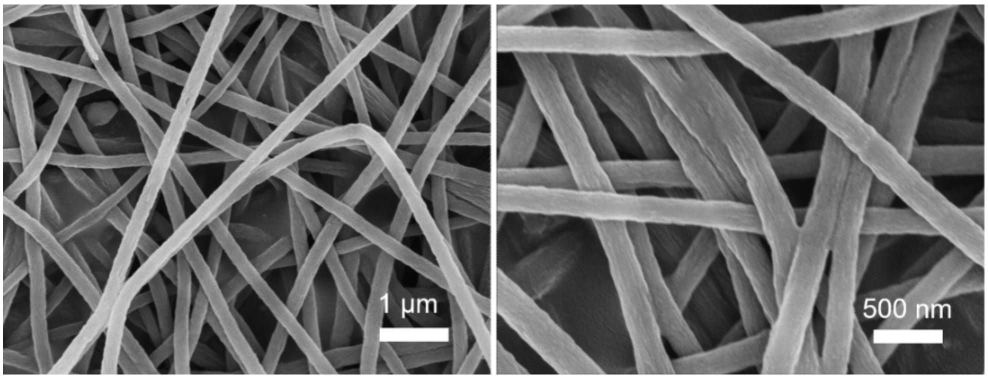

Fig. S4. SEM images of porous PAN/0.01PFSA.

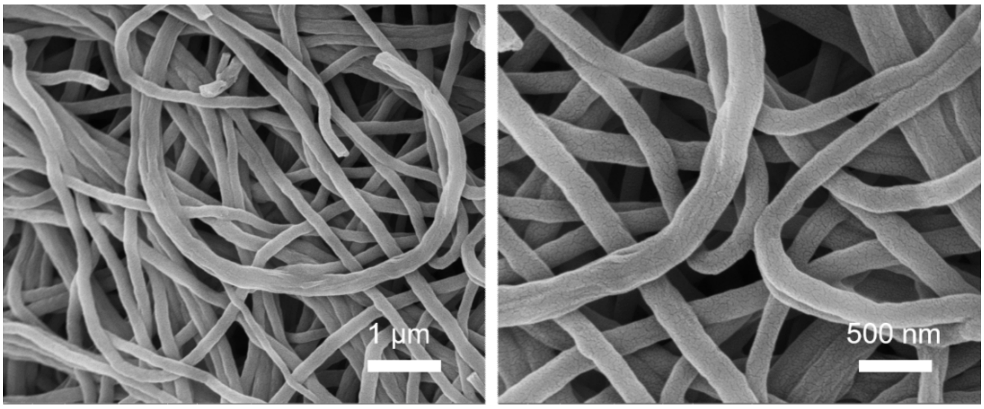

Fig. S5. SEM images of 0.01F-PCNFs.

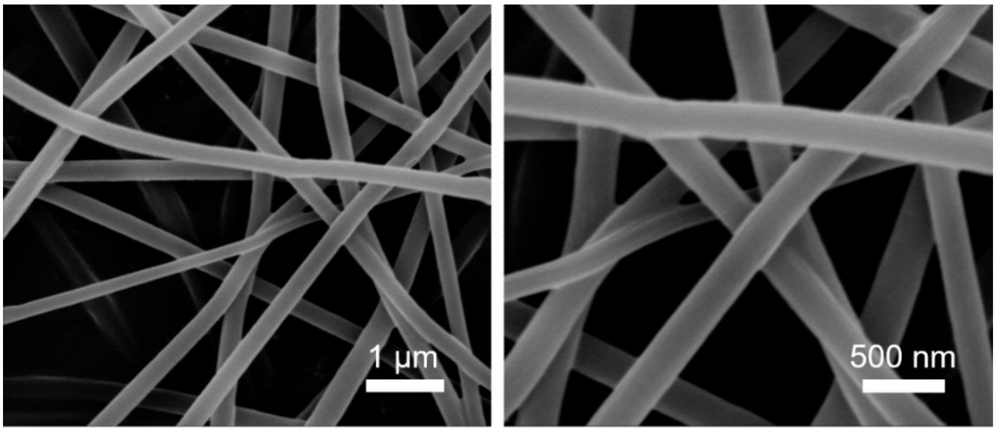

Fig. S6. SEM images of PAN/PVP/0.05PFSA.

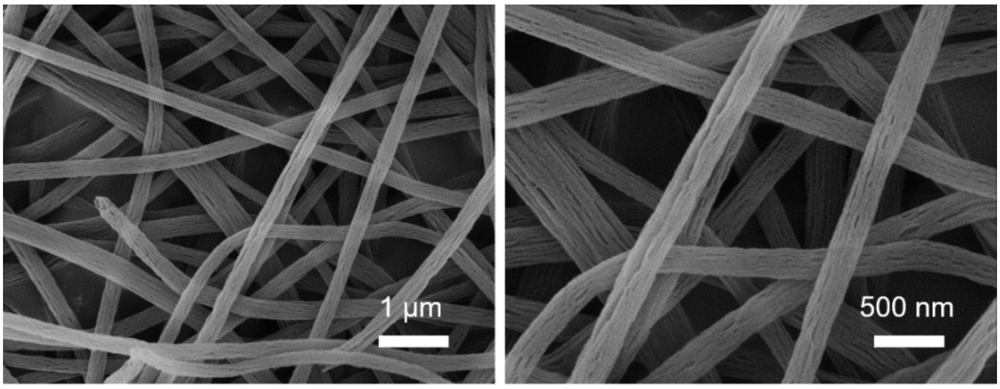

Fig. S7. SEM images of porous PAN/0.05PFSA.

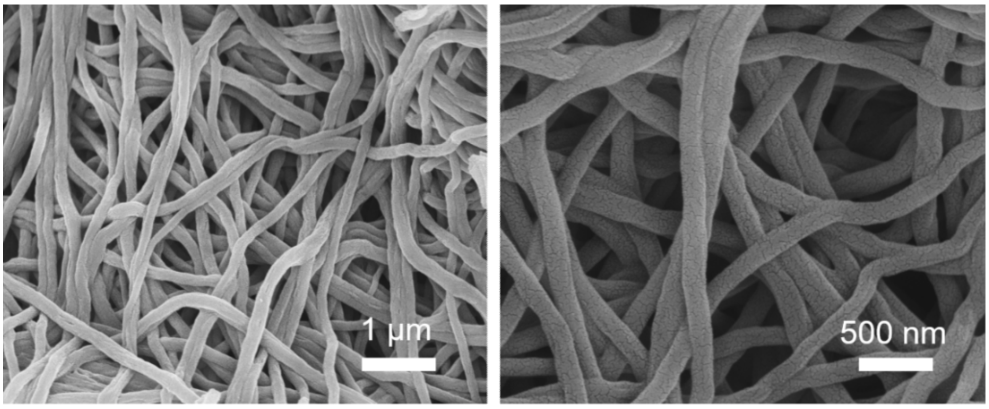

Fig. S8. SEM images of 0.05F-PCNFs.

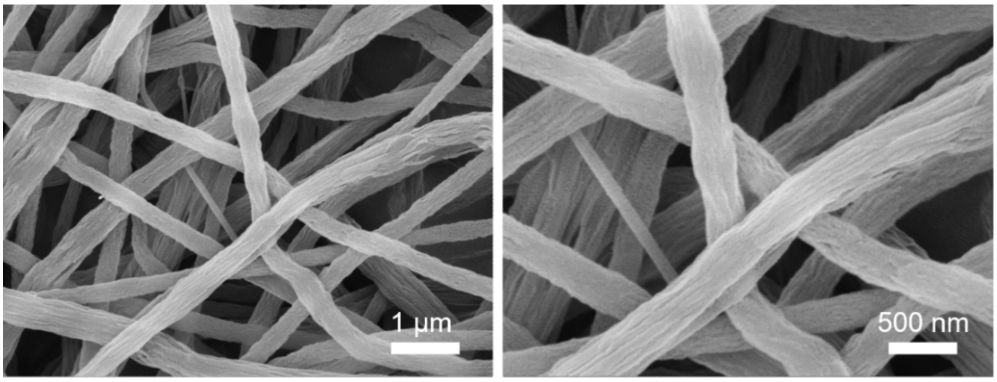

Fig. S9. SEM images of porous PAN/0.01PFSA-Li.

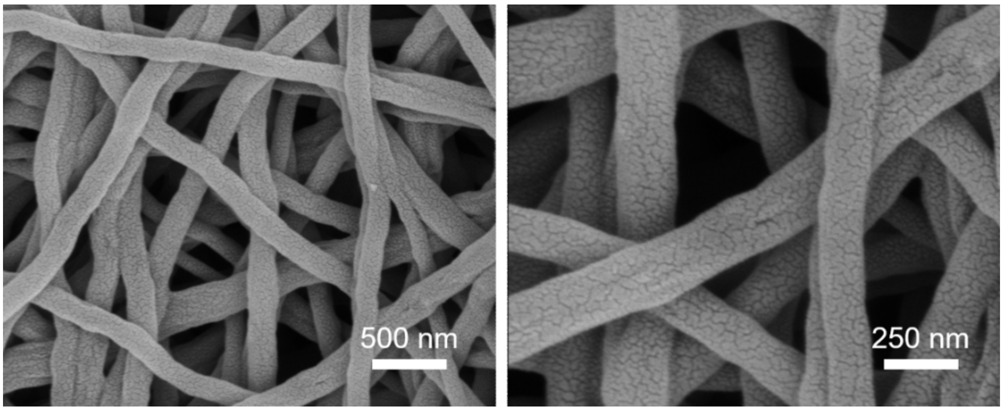

Fig. S10. SEM images of 0.01Li-F-PCNFs composites.

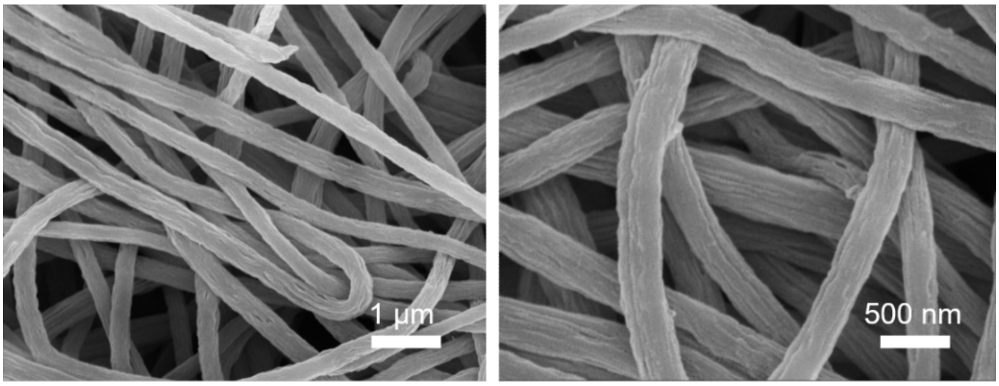

Fig. S11. SEM images of porous PAN/0.05PFSA-Li.

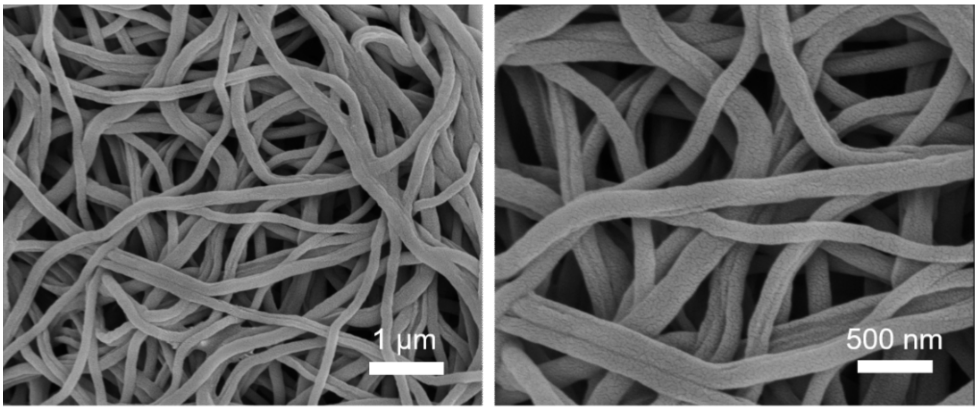

Fig. S12. SEM images of 0.05Li-F-PCNFs composites.

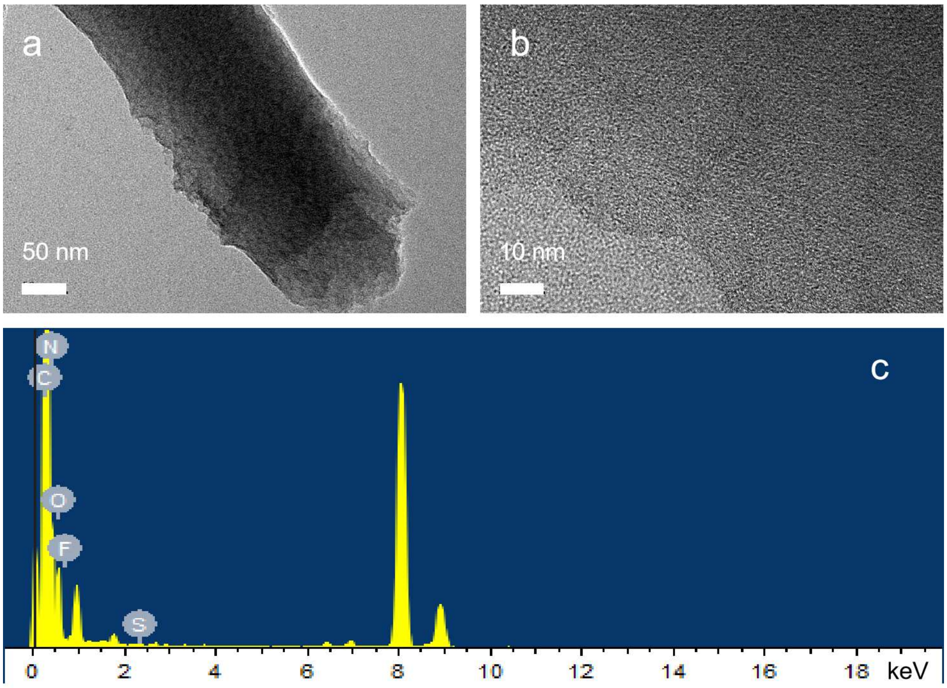

Fig. S13. (a, b) HRTEM images and (c) EDS of 0.1Li-F-PCNFs composites.

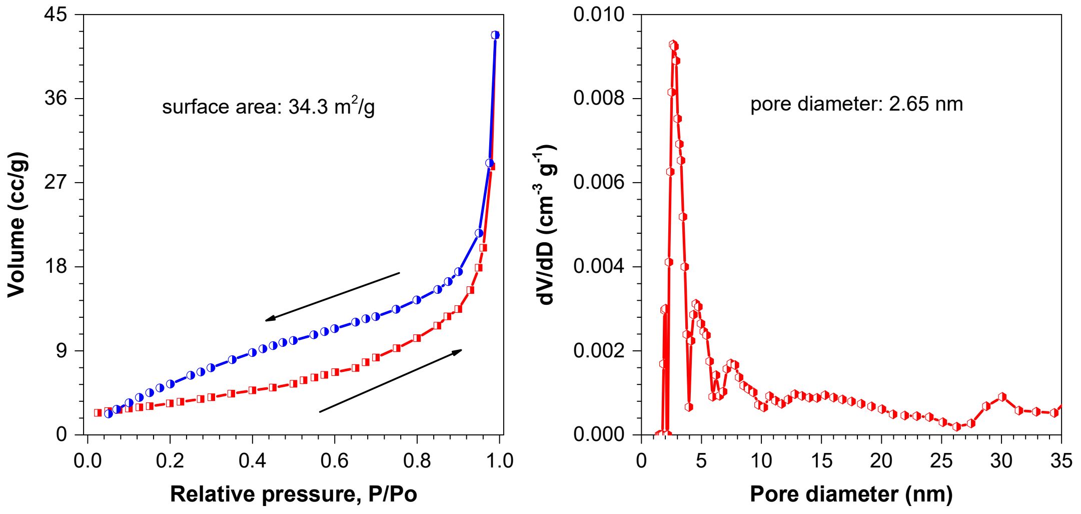

Fig. S14. Nitrogen adsorption–desorption isotherms of 0.1Li-F-PCNFs composites obtained at 77 K.

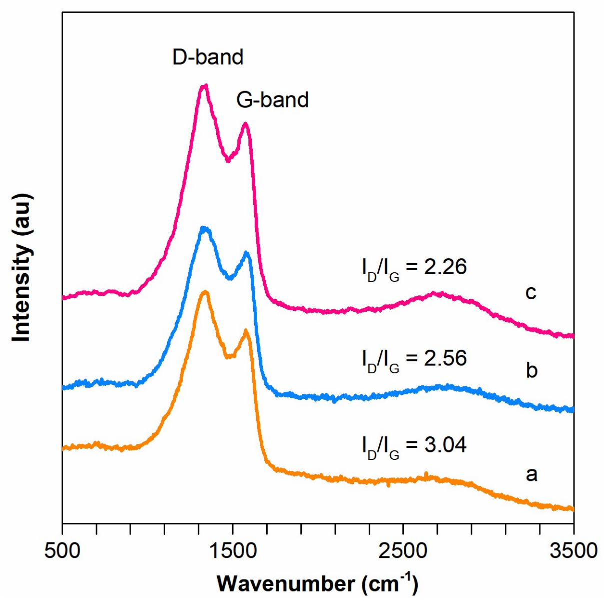

Fig. S15. Raman spectra of (a) 0.01F-PCNFs composites, (b) 0.05F-PCNFs composites, and (c) 0.1F-PCNFs composites.
